# Supplementary material for: The heterogeneity of dermal mesenchymal cells reproduced in skin equivalents regulates barrier function and elasticity
Source: EMBO Rep. 2026 Apr 1;27(9):2319–44. doi: 10.1038/s44319-026-00757-w (PMC13172354; doi:10.1038/s44319-026-00757-w)
Supplement: Supplementary file 3 — Expanded View Figures [file 44319_2026_757_MOESM3_ESM.pdf]

Expanded View Figures

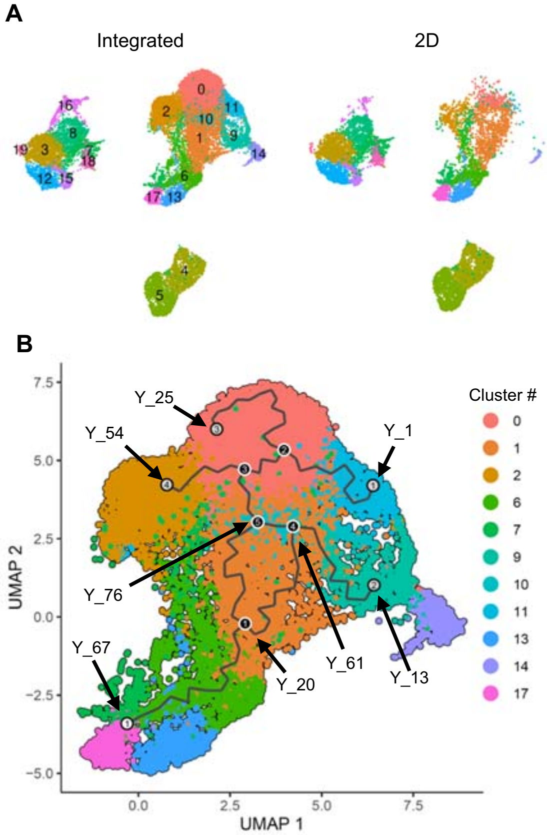

Figure EV1. scRNA-seq analysis of integrated in vitro samples.

(**A**) UMAP plot of scRNA-seq for in vitro human skin data, and distribution of all cells under each of the three conditions (2D, 3D_ED, and 3D_EDV). (**B**)Visualization of the pseudotime trajectories of fibroblasts clusters distinguished on the UMAP plot.

© The Author(s)

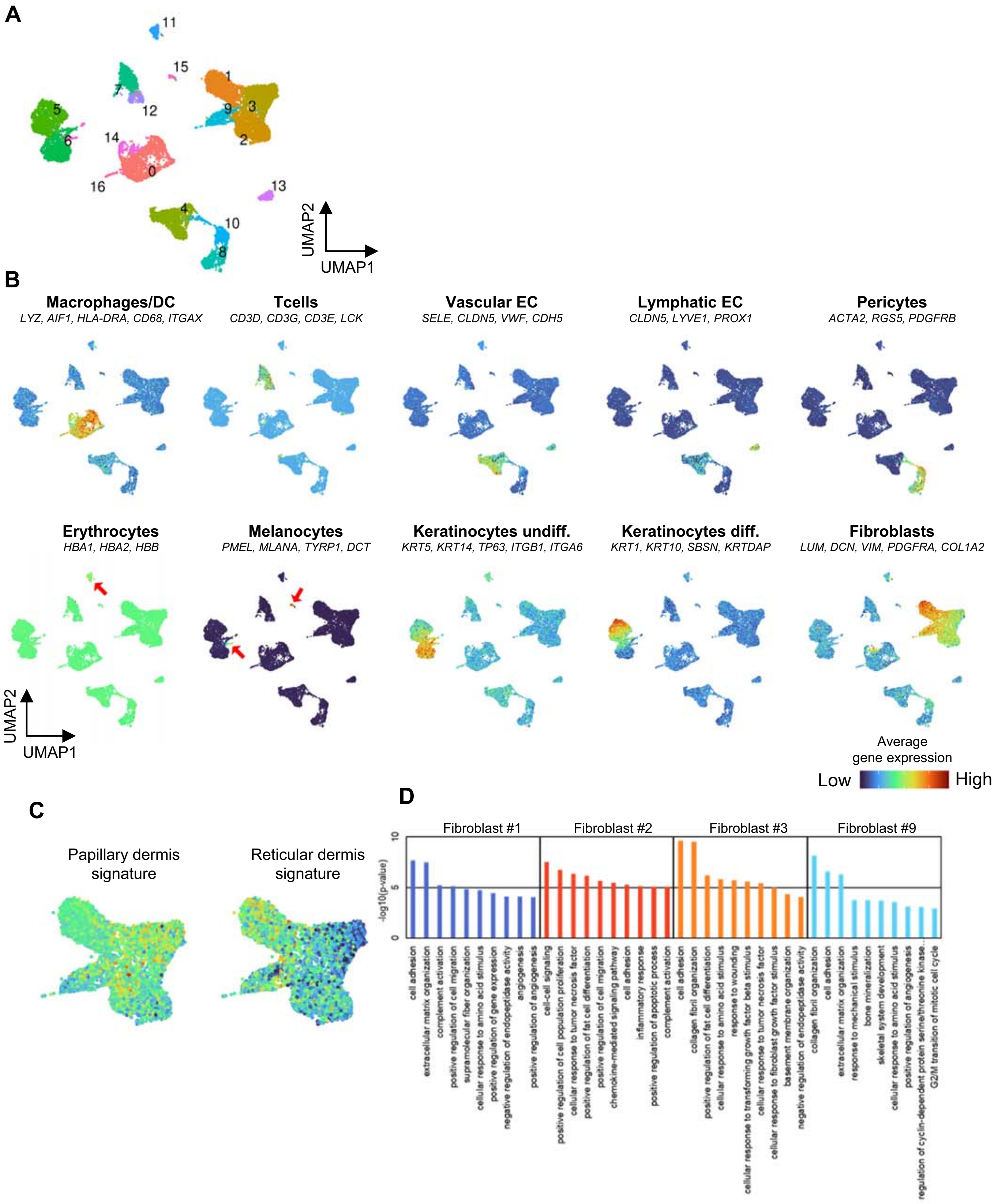
A
11
15
1
7
12
3
9
5
2
6
14
0
16
13
4
10
8
UMAP2
UMAP1
B
Macrophages/DC
LYZ, AIF1, HLA-DRA, CD68, ITGAX
Tcells
CD3D, CD3G, CD3E, LCK
Vascular EC
SELE, CLDN5, VWF, CDH5
Lymphatic EC
CLDN5, LYVE1, PROX1
Pericytes
ACTA2, RGS5, PDGFRB
Erythrocytes
HBA1, HBA2, HBB
Melanocytes
PMEL, MLANA, TYRP1, DCT
Keratinocytes undiff.
KRT5, KRT14, TP63, ITGB1, ITGA6
Keratinocytes diff.
KRT1, KRT10, SBSN, KRTDAP
Fibroblasts
LUM, DCN, VIM, PDGFRA, COL1A2
UMAP2
UMAP1
Average
gene expression
Low
High
C
Papillary dermis
signature
Reticular dermis
signature
D
Fibroblast #1
Fibroblast #2
Fibroblast #3
Fibroblast #9
-log10(p-value)
10
5
0
cell adhesion
extracellular matrix organization
complement activation
positive regulation of cell migration
supramolecular fiber organization
cellular response to amino acid stimulus
positive regulation of gene expression
negative regulation of endopeptidase activity
angiogenesis
positive regulation of angiogenesis
cell-cell signaling
positive regulation of cell population proliferation
cellular response to tumor necrosis factor
positive regulation of fat cell differentiation
positive regulation of cell migration
chemokine-mediated signaling pathway
cell adhesion
inflammatory response
positive regulation of apoptotic process
complement activation
cell adhesion
collagen fibril organization
positive regulation of fat cell differentiation
cellular response to amino acid stimulus
response to wounding
cellular response to transforming growth factor beta stimulus
cellular response to tumor necrosis factor
cellular response to fibroblast growth factor stimulus
basement membrane organization
negative regulation of endopeptidase activity
collagen fibril organization
cell adhesion
extracellular matrix organization
response to mechanical stimulus
bone mineralization
skeletal system development
cellular response to amino acid stimulus
positive regulation of angiogenesis
regulation of cyclin-dependent protein serine/threonine kinase...
G2/M transition of mitotic cell cycle

© The Author(s)

Figure EV2. Re-evaluation of in vivo samples through scRNA-seq.

In vivo skin single-cell analysis was carried out via methods described in previous studies(Solé-Boldo et al, 2020), (GSE130973, Data ref: Solé-Boldo et al, 2020). (**A**) A UMAP plot presenting single-cell transcriptomic data from whole human skin samples ($N = 5$). Each point signifies a single cell, with coloration based on unsupervised clustering executed using Seurat. (**B**) Mean expression of genes forming the papillary and reticular gene signatures applied to predict the dermal localization of fibroblasts within the four clusters. (**C**) A UMAP plot featuring the average expression of previously established cell type markers for distinguishing cell populations. Red denotes maximum gene expression, whereas blue represents minimal or non-existent expression of a specific gene set in log-normalized UMI counts. (**D**) The ten top significantly enriched GO terms within each fibroblast subpopulation, arranged by *P* value (hypergeometric test with Benjamini–Hochberg correction).

© The Author(s)

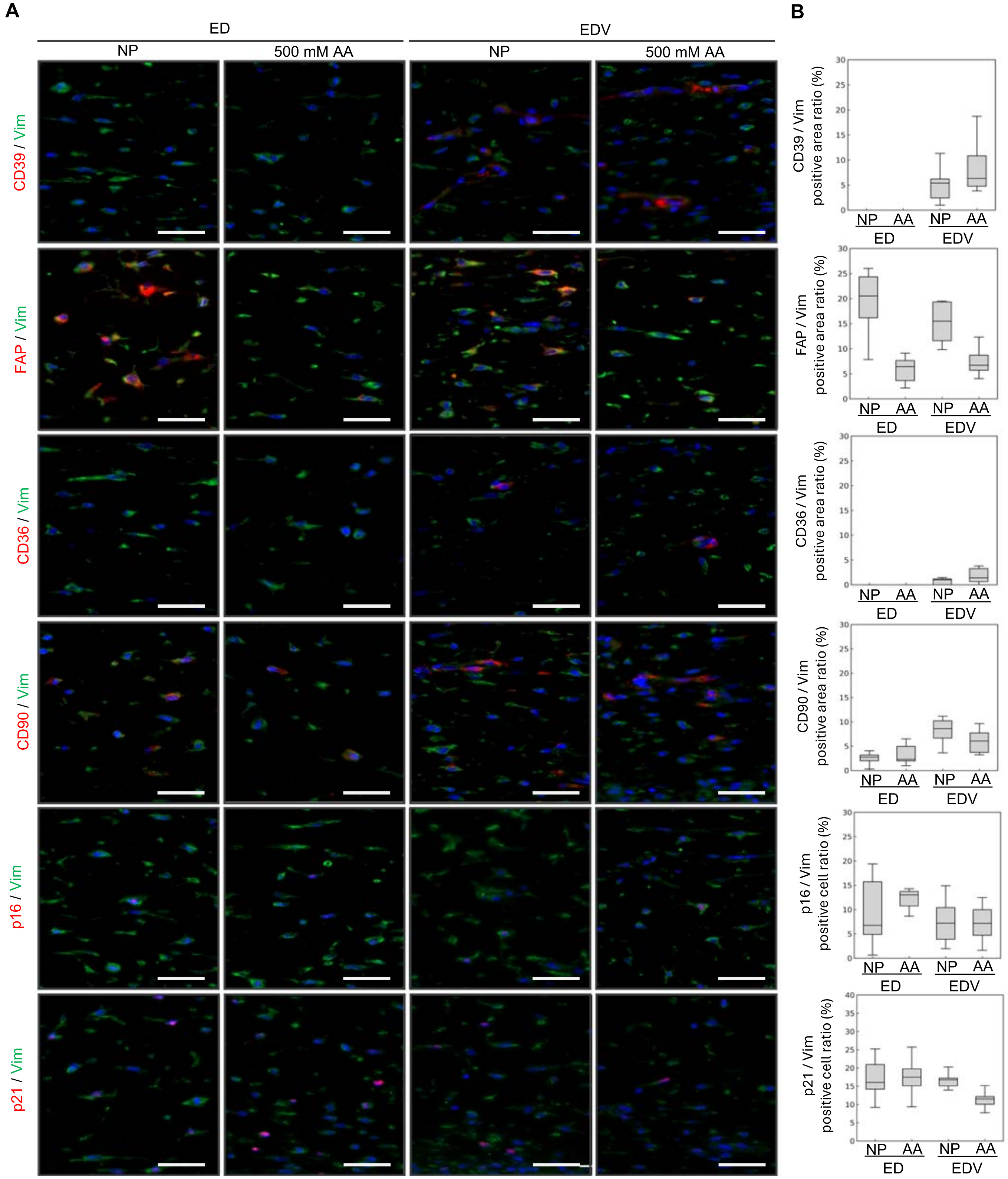
A
ED
EDV
NP
500 mM AA
NP
500 mM AA
CD39 / Vim
FAP / Vim
CD36 / Vim
CD90 / Vim
p16 / Vim
p21 / Vim
B
CD39 / Vim positive area ratio (%)
FAP / Vim positive area ratio (%)
CD36 / Vim positive area ratio (%)
CD90 / Vim positive area ratio (%)
p16 / Vim positive cell ratio (%)
p21 / Vim positive cell ratio (%)
NP AA NP AA
ED EDV

 © The Author(s)

Figure EV3. Immunohistological analysis of dermal mesenchymal cell distribution in the HSEs cultured under NP conditions.

(**A**) Immunohistochemical analyses of HSEs cultured under NP conditions with or without AA. Scale bar, 50 µm. (**B**) Quantitative analysis of the ratio of fibroblast marker-positive area. Box plots depict the median as the center line, the interquartile range as the box, and whiskers extending to 1.5 × the interquartile range. Outliers were omitted. $N = 9$, technical replicates.

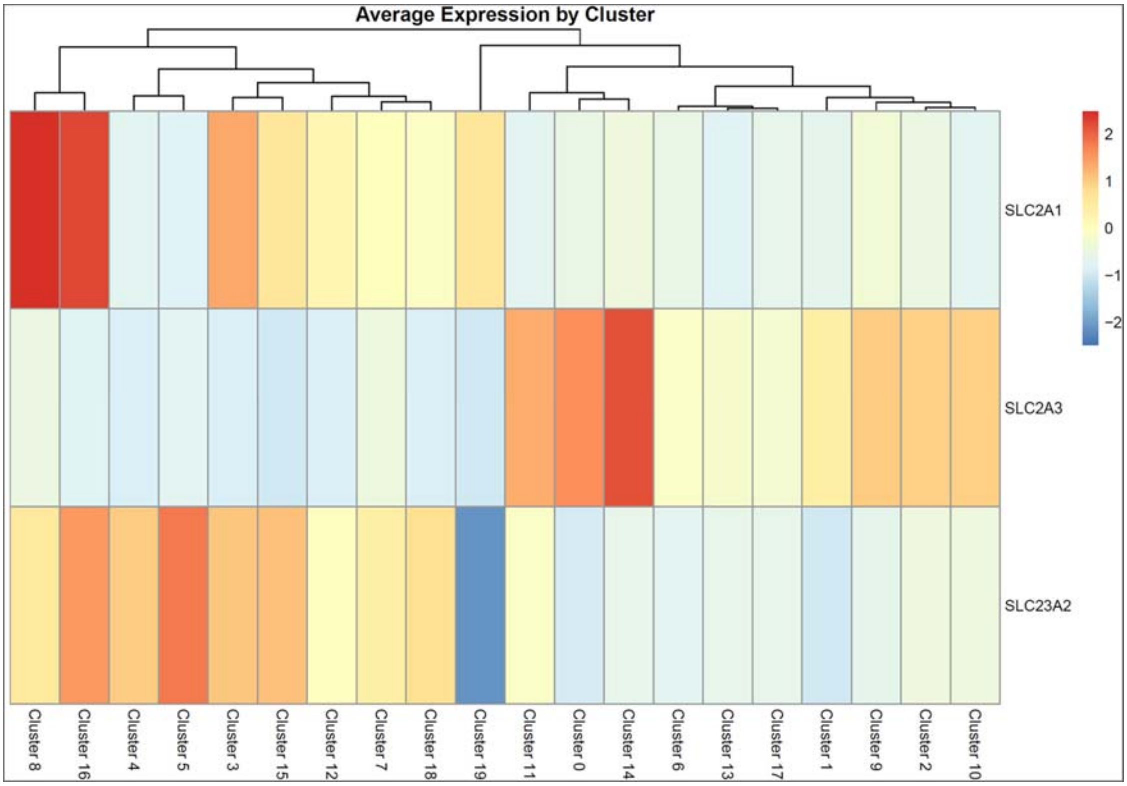

Figure EV4. Cluster-specific mean expression patterns of ascorbic acid and dehydroascorbic acid transporter genes.

Expression levels of ascorbic acid and dehydroascorbic acid transporter genes with a maximum mean expression above 0.01 are shown. Expression levels were scaled to Z-scores across clusters and visualized as a heatmap.

 © The Author(s)
